# Plant Therapeutics

**DOI:** 10.3390/plants11202720

**Published:** 2022-10-14

**Authors:** Juei-Tang Cheng, I-Min Liu, Szu-Chuan Shen

**Affiliations:** 1 Institute of Medical Research, Chang Jung Christian University, Tainan City 71101, Taiwan; 2 Department of Pharmacy and Master Program, Collage of Pharmacy and Health Care, Tajen University, Pingtung 90741, Taiwan; 3 Graduate Program of Nutrition Science, National Taiwan Normal University, Taipei 11677, Taiwan

Plants for therapeutics and the phytotherapy for disorders are the same thing in practice. Throughout history, humans have relied on nature material to meet their basic needs, particularly for the treatment of a wide range of diseases. Plants have thus served as the foundation of traditional medicine systems. Much plant research has been conducted in academia. Indeed, the information provided on crude extracts or active components of the plants comes from a variety of sources. Herbs are raw plant matters which are leaves, flowers, fruits, seeds, stems, and others, which may be in an entirely powdered form. Simple processes involve harvesting, drying of plant parts for preparation of phytochemicals. Increased awareness of intellectual property issues, combined with increased collaboration with the pharmaceutical industry, may result in the development of new drugs via this route. Therefore, many medicines are natural products or their derivatives [1]. This situation is likely to persist and increase for the growing prevalence of disorders that are resistant to modern medication around the world.

Ethnobotany is a subject that fills volumes of historical and biological texts but is largely ignored in contemporary texts. It is both an ancient way of life and a young and thriving scientific field. The word itself may provide the simplest definition of ethnobotany: *ethno* (people) and *botany* (science of plants). Thus, it is a study of how people from various cultures and regions use plants in their local environments. These uses can include food, medicine, fuel, shelter, and religious ceremonies in many cultures [2].

Ethnomedicine has a broad range in practices through two categories: *personalistic* systems, in which supernatural causes attributed to angry deities, ghosts, ancestors, and witches predominate, and *naturalistic* systems, in which natural causes predominate [3]. Where illness is explained in broad, impersonal in the traditional medical systems of native America, and parts of China, South Asia, Latin America. In African communities, the personalistic system appears to predominate (though not to the exclusion of naturalistic explanations). Naturalistic explanations predominate in Ayurveda, Unani, Khampo (Japan), and traditional Chinese medicine (CTM) without the exclusion of personalistic causation. Part of the belief in the later types is that the introduction of heat or cold into, or loss of, the body upsets its basic equilibrium, that is, the balance of humors, or the *dosha* of Ayurvedic medicine, or the *yin and yang* of Chinese medicine, and these must be restored if the patient is to recover. Therefore, the indigenous herbal medicine is known as a natural, herbal extract with little or no industrial processing used to treat disorders within local or regional use.

Plants play a major role in disorder management. Treatment is not limited to the sterile use of various leaves, roots, fruits, barks, grasses, and various objects such as minerals, dead insect’s bones, feathers, shells, eggs, powders, and smoke from various burning objects for the cure and prevention of diseases. If a sick person is given a leaf infusion to drink, they believe not only in the plant’s organic properties, but also in the magical or spiritual force infused by nature in all living things, as well as the role of their ancestors, spirits, and gods in the healing process [4]. The patient also believes in the power of the incantation and assists the handler in identifying the ingredients of the remedies given. In addition, rather than being a passive subject of therapy, the patient is an active participant in the treatment process [5]. According to the World Health Organization, despite improved access to modern medicine, a large segment of the human population still prefers traditional medicine, also known as alternative medicine. Therefore, placebo control is now critical in clinical trials of traditional medicine.

This Special Issue is going to publish the article(s) targeted on plants for therapeutics. Combination of plants and/or plant combined modern medicine are also popularly used in therapeutics. It is important to assess the renowned historical significance of products contained in the plant. We call the researchers to share the useful plant which is applied in each local market using the reliable model of disorders to indicate the effectiveness in therapeutics. Moreover, considering the high interest in plant-derived effectiveness in health as an emerging challenge for modern therapies, this Special Issue covers a wide variety of areas, aiming to contribute to the overall knowledge of medicinal plants from several aspects. These studies were evaluated by the journal’s regular review process, and we are pleased to publish the qualified results in this issue. In brief, we divided them into three sections: the first contains eight reports on plant crude extracts, the second contains seven reports on plant pure compounds, and the third contains two review articles.

In the section one, water-soluble extract of Safflower, also known as thorn flower, has been identified in crude extracts to inhibit human platelet aggregation induced by ADP in vitro. The findings were consistent with traditional Chinese medicine’s belief in promoting blood circulation and removing blood stasis [6]. Furthermore, using streptozotocin-nicotinamide (STZ-NA) induced type-2 diabetic rats, the aerial parts of *Eryngium longifolium* and the rhizome of *Alsophila firma* were tested for antihyperglycemic activity. For lyophilization, each plant was extracted with either water or ethanol. Then, for the first time, they confirmed the traditional use of two Mexican plants in animal model. Furthermore, aqueous (AE), methanolic (ME), and hexane (HE) extracts of *Acalypha monostachya*, another Mexican plant, were found to inhibit the growth of Human Tumor Cells in vitro. Another Mexican plant, *Inga jinicuil*, which is used in traditional medicine to treat gastrointestinal inflammation and infection, was tested in vitro. Three extracts of this plant’s bark demonstrated significant antibacterial activity. Moreover, the extract of the Korean plant *Lindera obtusiloba* was shown to improve endothelial dysfunction and ameliorate the plaque development in Hyperlipidemic ApoE-Knockout Mice, primarily by reducing vascular NADPH oxidase-induced ROS generation [7]. Furthermore, a water extract of fermented rice bran has been shown to protect against liver damage and intestinal injury in elderly rats fed a high-fat diet. Finally, this section included two herbal mixtures used in clinics of traditional Chinese medicine, Yi-Gan-San and Gan-Mai-Da-Zao. Yi-Gan-San, also known as Yokukansan in Japan [8] or Shun-Ning-Yi in Taiwan, is a commercial product of Sun-Ten Pharmaceutical company that contains seven medicinal herbs per 100 g of the final product: Bupleurum 2 g, Licorice 2 g, Chuanxiong 3.2 g, Angelica 4 g, Atractylodes 4 g, Poria 4 g, and Uncaria 4 g. Yi-Gan-San has been shown to reduce Aβand Tau expression in Drosophila melanogaster using the IMR assay and Western blotting analysis. It provided an effective treatment for neurodegenerative disorders in Alzheimer’s disease. Gan-Mai-Da-Zao also contained three major plants: blighted wheat (Fu Mai), licorice (Gan Cao), and jujube (Da Zao). Licorice (Gan Cao) was identified as the major herb in Gan-Mai-Da-Zao for antidepressant-like therapeutics in two rat models of depression-like disorders.

In the second section, Vescalagin isolated from Pink Wax Apple was shown to protect pancreatic β-cells from methylglyoxal-induced inflammation in rats. It suggested that Vescalagin could become a health food ingredient used to prevent diabetes. The phytochemicals and bioactivities of four major halophytic plants growing in central Saudi Arabia were then compared, including antioxidant, anticancer, and antimicrobial effects. *Lycium shawii*, a halophytic plant, has been suggested as the notable one based on phenolic and flavonoid content. Furthermore, three extracts (n-hexane, ethyl acetate, and ethanol) of *Acalypha arvensis* (Euphorbiaceae) were tested for antibacterial properties. In addition to corilagin, an active principle for the first time in this plant, chlorogenic acid, rutin, quercetin-3-O-glucoside, and caffeic acid were also discovered. Furthermore, researchers used traditional extraction methods such as the Soxhlet apparatus (SHS) and rapid solid–liquid dynamic extraction (RSLDE) to extract the principles contained in the plant *Salvia haematodes* L. AChE or BChE inhibitory activity was found in the SHN n-hexane fraction. Phytosterols, β-sitosterol and stigmasterol, have been proposed as active principles for use in Alzheimer’s disease therapy. The in vitro studies were used to develop by-products of the black bean fermented soybean sauce manufacturing process as new functional foods for anti-inflammation. Furthermore, the fruit bilberry (*Vaccinium myrtillus* L.) and yeast β-glucan have been shown to help with diabetic complications. The fast-dissolving films were created to combine both. The new product provided an improved fast dissolving time and good water vapor barrier properties, suggesting its success as a novel film for packaging dry powdered pharmaceuticals.

Two review articles were arranged in the final section. One gathered information on the benefits of *Moringa oleifera* Lam., also known as the drumstick tree, in renal diseases. This review article used a meta-analysis of the database from 2011 to 15 June 2021. It was proposed that this plant reduces several pathological factors associated with kidney disease, such as inflammation and oxidative stress. Another review article suggested that natural products or active principles from plants could help treat Triple Negative Breast Cancer (TNBC) by impeding the Wnt/β-Catenin Pathway. The authors mentioned 26 different types of active principles from the plant that are useful for TNBC. This review article also introduced three herbal products including herbal mixture and the useful plants [9]. In the conclusion, it is suggested that natural bioactive compounds be evaluated in clinical trials.

This Special Issue differs from the traditional hard or soft cover book concept. This approach will make this topic more accessible to a broader range of readers. It gives us great pleasure to write a foreword for this lovely, multi-authored international publication on a unique subject. We wish this Issue great success and hope to see a regular series develop from this point forward. We also want to thank all the contributors for their outstanding efforts in making this Issue a success.
